# Supplementary material for: Genome-wide identification and characterization of the OFP gene family in Chinese cabbage (Brassica rapa L. ssp. pekinensis)
Source: PeerJ. 2021 Mar 5;9:e10934. doi: 10.7717/peerj.10934 (PMC7938782; doi:10.7717/peerj.10934)
Supplement: Table S1 [file peerj-09-10934-s001.docx]

**Table S1.** The synonymous (dS) and nonsynonymous (dN) nucleotide substitution rates for orthologous *OFP* gene pairs between *B. rapa* and *A. thaliana.*

| *BraOFP* | *AtOFP* | dN | dS | dN/ dS |
| --- | --- | --- | --- | --- |
| BraA02.OFP1.a | *AtOFP1* | 0.201 | 0.7418 | 0.2709 |
| BraA03.OFP2.a | *AtOFP2* | 0.1495 | 0.3504 | 0.4267 |
| BraA04.OFP2.b | *AtOFP2* | 0.1588 | 0.3531 | 0.4498 |
| BraA05.OFP2.c | *AtOFP2* | 0.1079 | 0.3191 | 0.3381 |
| BraA10.OFP3.a | *AtOFP3* | 0.1647 | 0.4128 | 0.3989 |
| BraA03.OFP4.a | *AtOFP4* | 0.6003 | 55.3964 | 0.0108 |
| BraA09.OFP4.b | *AtOFP4* | 0.1607 | 0.3983 | 0.4035 |
| BraA01.OFP5.a | *AtOFP5* | 0.0776 | 0.2581 | 0.3006 |
| BraA03.OFP5.b | *AtOFP5* | 0.1085 | 0.2586 | 0.4196 |
| BraA08.OFP5.c | *AtOFP5* | 0.1097 | 0.2754 | 0.3982 |
| BraA06.OFP7.a | *AtOFP7* | 0.1895 | 0.5787 | 0.3274 |
| BraA07.OFP7.b | *AtOFP7* | 0.134 | 0.4551 | 0.2944 |
| BraA02.OFP8.a | *AtOFP8* | 0.1359 | 0.3915 | 0.3472 |
| BraA10.OFP8.b | *AtOFP8* | 0.1374 | 0.3302 | 0.4163 |
| BraA02.OFP10.a | *AtOFP10* | 0.0894 | 0.3569 | 0.2505 |
| BraA10.OFP10.b | *AtOFP10* | 0.0788 | 0.7186 | 0.1096 |
| BraA01.OFP11.a | *AtOFP11* | 0.1481 | 0.3865 | 0.3832 |
| BraA09.OFP12.a | *AtOFP12* | 0.0863 | 0.4597 | 0.1878 |
| BraA10.OFP12.b | *AtOFP12* | 0.1403 | 0.602 | 0.2331 |
| BraA02.OFP13.a | *AtOFP13* | 0.075 | 0.7022 | 0.1068 |
| BraA10.OFP13.b | *AtOFP13* | 0.0801 | 0.7287 | 0.1099 |
| BraA02.OFP14.a | *AtOFP14* | 0.1918 | 0.7066 | 0.2714 |
| BraA07.OFP14.b | *AtOFP14* | 0.1195 | 0.5988 | 0.1996 |
| BraA04.OFP15.a | *AtOFP15* | 0.1019 | 0.2555 | 0.3989 |
| BraA05.OFP15.b | *AtOFP15* | 0.1067 | 0.3803 | 0.2805 |
| BraA05.OFP16.a | *AtOFP16* | 0.1597 | 0.5616 | 0.2844 |
| BraA09.OFP18.a | *AtOFP18* | 0.1654 | 0.4449 | 0.3718 |
| BraA04.OFP19.a | *AtOFP19* | 0.0659 | 0.5665 | 0.1163 |
| BraA05.OFP19.b | *AtOFP19* | 0.0856 | 0.6811 | 0.1257 |
