# Supplementary material for: Genome-wide identification and characterization of the OFP gene family in Chinese cabbage (Brassica rapa L. ssp. pekinensis)
Source: PeerJ. 2021 Mar 5;9:e10934. doi: 10.7717/peerj.10934 (PMC7938782; doi:10.7717/peerj.10934)
Supplement: Table S2 [file peerj-09-10934-s002.docx]

**Table S2.** The duplicated types of *OFP* genes in *B. rapa*.

| Gene name | Duplication type | Gene name | Duplication type |
| --- | --- | --- | --- |
| BraA02.OFP1.a | Dispersed | BraA10.OFP10.b | WGD / Segmental |
| BraA03.OFP2.a | WGD / Segmental | BraA01.OFP11.a | Dispersed |
| BraA04.OFP2.b | WGD / Segmental | BraA09.OFP12.a | WGD / Segmental |
| BraA05.OFP2.c | WGD / Segmental | BraA10.OFP12.b | WGD / Segmental |
| BraA10.OFP3.a | Dispersed | BraA02.OFP13.a | WGD / Segmental |
| BraA03.OFP4.a | Dispersed | BraA10.OFP13.b | WGD / Segmental |
| BraA09.OFP4.b | WGD / Segmental | BraA02.OFP14.a | WGD / Segmental |
| BraA01.OFP5.a | WGD / Segmental | BraA07.OFP14.b | WGD / Segmental |
| BraA03.OFP5.b | WGD / Segmental | BraA04.OFP15.a | WGD / Segmental |
| BraA08.OFP5.c | WGD / Segmental | BraA05.OFP15.b | WGD / Segmental |
| BraA06.OFP7.a | WGD / Segmental | BraA05.OFP16.a | Dispersed |
| BraA07.OFP7.b | WGD / Segmental | BraA09.OFP18.a | WGD / Segmental |
| BraA02.OFP8.a | WGD / Segmental | BraA04.OFP19.a | WGD / Segmental |
| BraA10.OFP8.b | WGD / Segmental | BraA05.OFP19.b | WGD / Segmental |
| BraA02.OFP10.a | WGD / Segmental |  |  |
