# Supplementary material for: Genome-wide identification and characterization of the OFP gene family in Chinese cabbage (Brassica rapa L. ssp. pekinensis)
Source: PeerJ. 2021 Mar 5;9:e10934. doi: 10.7717/peerj.10934 (PMC7938782; doi:10.7717/peerj.10934)
Supplement: Table S3 [file peerj-09-10934-s003.docx]

**Table S3.** The physicochemical parameters and subcellular localization of OFP proteins in *B. rapa*.

| Protein name | Length  (aa) | Molecular weight (KD) | PI | Instability index | GRAVY | Subcellular localization |
| --- | --- | --- | --- | --- | --- | --- |
| BraA02.OFP1.a | 263 | 29.39 | 10.28 | 73.43 | -0.60 | Nucleus |
| BraA03.OFP2.a | 308 | 35.46 | 9.89 | 51.86 | -0.94 | Nucleus |
| BraA04.OFP2.b | 324 | 37.10 | 9.79 | 64.96 | -0.99 | Nucleus |
| BraA05.OFP2.c | 316 | 36.41 | 9.78 | 57.8 | -0.98 | Nucleus |
| BraA10.OFP3.a | 342 | 38.44 | 10.05 | 63.4 | -0.90 | Nucleus |
| BraA03.OFP4.a | 113 | 13.75 | 6.61 | 64.36 | -0.64 | Nucleus |
| BraA09.OFP4.b | 301 | 34.51 | 10.42 | 67.45 | -0.93 | Nucleus |
| BraA01.OFP5.a | 327 | 38.06 | 9.57 | 46.98 | -1.08 | Nucleus |
| BraA03.OFP5.b | 301 | 35.30 | 9.6 | 47.67 | -0.92 | Nucleus. |
| BraA08.OFP5.c | 321 | 37.36 | 9.91 | 49.2 | -1.01 | Nucleus |
| BraA06.OFP7.a | 403 | 44.83 | 9.9 | 81.72 | -0.46 | Nucleus  Chloroplast |
| BraA07.OFP7.b | 315 | 35.72 | 9.97 | 96.2 | -0.77 | Nucleus |
| BraA02.OFP8.a | 206 | 23.78 | 9.41 | 61.51 | -0.58 | Nucleus |
| BraA10.OFP8.b | 213 | 24.53 | 9.08 | 63.49 | -0.67 | Nucleus |
| BraA02.OFP10.a | 162 | 18.24 | 8.81 | 57.02 | -0.56 | Nucleus  Cell membrane  Chloroplast. |
| BraA10.OFP10.b | 88 | 10.31 | 5.41 | 45.54 | -0.66 | Nucleus |
| BraA01.OFP11.a | 214 | 23.89 | 5.23 | 54.79 | -0.54 | Nucleus |
| BraA09.OFP12.a | 227 | 25.08 | 4.81 | 55.38 | -0.47 | Nucleus |
| BraA10.OFP12.b | 300 | 33.44 | 5.59 | 51.39 | -0.49 | Nucleus |
| BraA02.OFP13.a | 223 | 24.81 | 4.58 | 62.22 | -0.49 | Nucleus |
| BraA10.OFP13.b | 262 | 28.35 | 4.77 | 57.88 | -0.31 | Nucleus |
| BraA02.OFP14.a | 295 | 34.17 | 9.2 | 79.8 | -1.05 | Nucleus |
| BraA07.OFP14.b | 286 | 32.77 | 9.08 | 57.32 | -0.94 | Nucleus |
| BraA04.OFP15.a | 261 | 29.01 | 4.72 | 56.05 | -0.49 | Nucleus |
| BraA05.OFP15.b | 270 | 30.02 | 4.56 | 63.94 | -0.44 | Nucleus |
| BraA05.OFP16.a | 237 | 26.41 | 5.44 | 67.99 | -0.48 | Nucleus |
| BraA09.OFP18.a | 258 | 29.68 | 6.97 | 73.83 | -0.42 | Nucleus |
| BraA04.OFP19.a | 186 | 21.00 | 9.46 | 53.53 | -0.55 | Nucleus |
| BraA05.OFP19.b | 186 | 20.88 | 9.11 | 56.54 | -0.48 | Nucleus |
