# Supplementary material for: Genome-wide identification and characterization of the OFP gene family in Chinese cabbage (Brassica rapa L. ssp. pekinensis)
Source: PeerJ. 2021 Mar 5;9:e10934. doi: 10.7717/peerj.10934 (PMC7938782; doi:10.7717/peerj.10934)
Supplement: Table S4 [file peerj-09-10934-s004.docx]

Table S4 The sequences of all *BraOFP* genes.

>BraA01g010340.3C

ATGATGATGAGATGGGGAAGAAAGAGACCTGTTTCTTCTTCTTCTTCTTATGGGTTGTCTCGTGCTCATCCTGTTTCTTGGTTTTCAAAGTTGAGTGGTT

CTTCTGACTTGAAACCTGCAAAAGAGAAGAAGCAAGATGATGAAAAATCTAGCAAAACGTTACAGCGAGTATCAGTAGAAAAGGAGAACGCTGCAACAAG

GTCAGCTAATATGGAGTCAACCGACAAGTTCGAAGAGATAATGAGCAGTGTGCGGAAGAAAGTAAGAGATTGCCAAAGAGAGACGTGTGGCTTTCTGAAA

GTAGAGGCAATGGATAGAGACAAAGGAACTGTAAGAATTCAGGTGAACAGAGAGAAGCAGAGATGTGAGAGACGTGACCAAAGGCTTCTCGAACAAAAGC

CAAAGAGATCAGAACAAGACGCAGAGATCAAGGCCAAAAATCCAGCGAGAAGGATAGGTACAGGGAGTTACAGCAGAAACGATTCTGTGATTCTTGGTCA

TACCGAATCAAAACCGGCTCATCAGTGGCAAAAGCCCAAGGATATAAAACTACGAGAAACGAAGTTGAAGGCTGACCAACAGAGGAAATCTCTGTACCTA

AGAAGGGAGCTTAACAGATTAGGAACAAAAGAAAGCAACAAGGTTAGAGTTTTTTCACCAAGAGCATCTGAAAAATGCAGAGTTAAAGCTATTGAAGACT

TGAAGAAGGCTAAACTTAGAGCACGGGAACAAGAACACGAGCTTGTGAAGGAAGAAGCAGATGGAGGAATGGAGAACGAAAGCTTTGCATTAGTGAAATG

CTCGAGCGATCCTCAGAGGGATTTTAGAGATTCAATGATTGAGATGATCATGGAGAATGGCATCAACCACCCTGAAGATCTCAAAGGGCTTCTGGTTTGT

TACCTCAGACTCAATAGCGATGAATATCATGACATGATCATCAATGTGTTTCAGCAAGTGCATCACGATTTAAATTTTCATTAG

>BraA01g021760.3C

ATGTCTACATTTCTAAGGAAGAAGCTACACCTATGCTTCTCCTCCTCCGGTGTTGTATCACCGTCGATTCCTTCTTCCCCGATCGTCGTACCCAATCACA

ACCCACCATCTAATCATCATCATCACCACACTCCCTCAATCTTCATCAACAACTTCAACTCTCTCTACGATCACCTCTCTGTTTCCTCTCCTCTCCACCG

TAACGATAAATTCACCTCCGTCGCCGCCGCATTGACCACTCCCAAATCCGGCGACACTGGATCCAGTTTGTTCACCGGTGGATTGCATCCTTCTCCGCGT

AGAGCTGACGACGAAGACGACGAAGAAGATGAAGATGACGAAGATGAAAGTTACACCGTAGTGTCAAAGCTTTTAAGCGACGGAACGGCGATTATGAAGC

AAATTGACTCACCGGATCCGTGCAGAGATTTCGGAAGGTCGATGAGAGAGATGGTGGAAGCTAGAGATCCGACGAGAGACGACGTCTCCGATAGAGAATA

CTTGAACGAACTGCTCTTCTGTTACCTCTCTTTGAATCCTAAACATACTCACAAGTTCATCGTCTCTGCTTTCGCAGATACACTTCTCTGGCTACTTTCT

CCGTCGTCATCGCCGGAATATTATTCTTTATCCCAATCGATTTAA

>BraA02g000100.3C

ATGGGTAATTATCGGTTTAAGCTATCGGAATTAGTCCCTAACGCATGGTTTTACAAACTGAGAGACATGGGGAAATCAAGGAAAAAGCAACATAACAGCA

CGGTGTCTCTTTCTCCCATCAAGAAACACCAGCCAGTTCCTACTCCTACTCCTACCACAGCACGCAGTCCTAGACTTCCTCTTCGTCGCTCGTCTTCTTA

CTATGCAAGGCCCAGAAAGTCTGTCCGCAACCGCGCCACGACCGTCTCCTCTGCTCCTCCGAAGCAAGTCACTGAGTCTTTCTCACCACCCGAGTTTCGC

TCTGATCAACTACTCATCCCTGAGGAGAGCTCCTCGCACAGCCCTTGCAGCAGCTGCGTTAGCTCCAAAGCCGCAACTCTTACGCCCCCTCCGGAACTCG

ATCTCCATCCGATCATCACCAAACCCACCACCACCGTGCGCAAAACGGCTGTGAGTACTTCTCCCGCGGGTGTGAGACTTAGACTGCGCTCGCCAAGGAT

CTCCACGAGGAAAACTGCGAGCAGCGGCGGCGCACGGATAGCCGGCACATCGGCGACTGCGAGTTCGCGGCGGAGTAGGGCGGTGGTGAAGGCGTCGGTA

GACCCGAGGAGAGATTTTAAGGAGTCGATGGAAGAGATGATCACAGAGAACAAGATAAGAGAGTCCAAGGATCTAGAGGAGCTTCTAGCTTGCTATCTCT

GTCTCAATTCTGACGAGTATCACCACATTATCATCAATGTCTTCAAGCAAATCTGGCTTGATCTTAATCTTTCACCCCATCTATCTTTATAA

>BraA02g001490.3C

ATGGGGAAGACGAAGCTCTCATCTCTGTTCAGAGGCGGAGCGGGAAGATTACTAACCGCTCCTCTCTGCTCCAATGCCAAAACACTGTCGTTCCGAGTCG

AAGACGACATGATCAAGACCGTTAACTCGGTCTTCTTCGATGATATATTAGAGGCCGCAACGCCCGAGTCATGGTTCACGAACTCGTCCGAGACGGCGAG

TCACTCAACCGAGTCGGACCAAGACCTCGACGCGGAATCGCTTGAGACGGTCGTTAGAGGAGTGGTTAGATCGGAGCGGCTGTTCTTCGACCCGGGAGCC

ACTAGCTCGATCGTGGAAGAGACCAACGAGAAATCGATCGAGGAGATCAGCGTCTCGGTGGCGATGGAGTCGGAGGATCCGTACGGCGATTTCCGGCGAT

CGATGGAGGAGATGGTGAGGAGCCACGGCGAGCTGGCTAAGGACTGGCGGAGCTTGGAGTCGATGCTGGAGTGGTACTTGAGGATGAACGAGAGGAGGAG

CCGCGACGTGATCATCAGCGCTTTCGTTGATCTCGTCTCCGGACTCTCTGATTCGGTGTCGGACTCGGGTCGTTACTCCACCGCCGTGTCGTCTTTGCCG

TCGTCGCCGTTATGTTCGTTATCGTCTAAAGGTGAGACGGAAACTGAAGAAGAAGAGAGACGGAGCCGTTAA

>BraA02g008340.3C

ATGGACAAAAGAATGAGGCTCAGGGTTCCAAGCATAGTCAGGTCATCTCTAAACTCTTGCCGATCACGAGGTCTATACGACGTCTTTGACACTTCTGCTG

TTGCAAGCCACACCACCTCTTCCGAGAGATTCTTCCTTACCAAAGCTCCACGCGTGGACCGTCACAAACCTAAGCCTTATGCCTTCCCTCCAAACCCTTT

CTACGAAGGAAGCCGTTCCTTTCGAGATATAAGGAAGAAGATTAAGACAAAGAGGAAGCAGAGATCATCTCAGTTTGGTTCTGATCCTCTTCTTACCTCT

AGTTTTAAGTCAAGTGGGTCTTGGTGCTGGTCGTGTAGCGAGGAAGAAGAAGAGAGTGACGATAGAGATACACTCGTTTCTTCTAGAAGCTTCTCCTCGG

ACTACTCTAAGGGGGAGAGTTTTGCGGTGGTGAAGAAGTCACATGATCCGTATCAGGATTTCAGGAAGTCAATGGTGGAGATGATTGTTGAGAGACAGAT

CTTTGCAGCGGCCGAGCTACAACAGCTTCTTCAGTGTTTTCTATCGTTGAACTCTCTTCAACACCACAGTGTTATAATCCAAGTGTTCTTAGAAATATAT

GCCACCTTGTTCTCTACCTAA

>BraA02g009640.3C

ATGCCTTTGAAGAAGAAGATGAAATATACACTCAAAACCATACTCAAGCCCATTTTCATGCCCTGTGGTTGCGGCTCCACCGTCCCGCCTCCATCTCACA

GCCACTACACCCCAGGACTACCGGTTTCTCCCACGGTGTTACGCAGCCCATGTCCTAAGATTGATGAGAGCGTCGCAATGGCTAAGGAATCATCCAATCC

ATTCGAAGATTACAAGATGTCTATGAATCAGATGATCAAGGAGAGAGATATTAAAACAGATGATGATCTTAAGGAGCTTCTTAGGTGTTTCCTGGACATA

AACCCTCCTCCTCAACACAATCTCATCGTCCGGGCTTTTGTGGACGTCTGTTCAGTTCTTGGGCCACCACACGACCGCCGTGGAAACTCACTTGGCAGAT

TGCTTCGTCTTTATGTTAATCCTGTTGATGATAATGATGATAACTCACACCAAACCTCTTCATTTAAGATGAGAAACGGATCCCCATGA

>BraA02g026230.3C

ATGCCAAACCCAGTGCAGCAATCGGTGTATGGTTACCTGTCAAAGATAAAAAGAGAGGCAGGAAAGCTGCAGTCGTCGTCGTCCAAATCATTGTCATCAT

CCAAGAAGTGGGTGCTTAGCTATAAGCTCCACAAAAAGCTCTCCTCCTTAACGAATGTACGACGTCCCAGCAAGACCAGCAGATTCAACGACGACCACCC

CCAGGATCCCGGTCAGGCCGCCACCTTGTCCGATATCGACCGTTTTCTCGAGGAAAACTTCAAATCCCTCTGCATCAGAGATGGCGGCGAAGAGGGGGAG

GAGGACCACCGTCGGAGGATGAGCAAAAAGAAGGAGAAAAGGGATCAGTCCTCGGATGACTCATCCGACACTGATGAAGACGACTACTATCGTCACAGAT

TCCAGAGGACATGGGAACCTGCGGTCTACGACTCCCCCAAACTGCCAGATCCGCGGAGGAGAACAGGGAGACTATCTCCACCACCTGGATCATCGGAAGG

TACGCCCAGCATGTACACAACGCCGGAGGAGGAGAGACGGTCGTTGAGGTCCCACTCATCGAGCTTGGTGCTGCCTGAGAACTGCATCGCGGTGCTGAGA

TGCACAGAAGAGCCTCAGGAGGATTTCAGGAGGTCGATGGTGGAGATGATGGAGTCCAAGCTAGGAGGGGGCGAGGTGGACTGGGACTTGATGGAAGAGC

TACTCTTCTGCTATCTCGATCTCAACCACAAGAAATCACACAAGTTCATACTCAGCGCATTCGTCGATCTCATCATCTCTCTCCGTGTCAAGGAGAAGAG

GGTCACCAGGAAAAGCCTTGAGATGTCGCTCAGTACTCGCGCCGCCAGGGACAGGTTTAGTAAGAGGAGCTATGCCTCTCGAAACTAG

>BraA03g016190.3C

ATGGGAAATTACAAGTTCAAAATTTCAGATATGATCCCAAATGCATGGCTGCACAAGCTCAAAGACATGACTAAGCAGTCTAAACCCAAACACAAACCTT

CTTCTTGTTCCTCAAACACTTGTAATAAGAAGAAACCCTCCTCAGATTCTCCTCTTCTTCATCACTCTTCAACCTTTCGTTTTTCCAACAGCTTAGTACC

AAACAATCCTCACCGTAACTCACCAAGAAACTCTCTTTACACCAAAAGGAAGAGTAAGAGAAAGACACTTTACAAGTCATCTCTTAAACCAGTCACTCCT

TTTGCATCTGAAAGTTCCACTCAGAGCTTGTTTCCAGCTCTTGAAAGCTCCCCTGAGTATTTTTTATTTAGTTTCTACGGAAAAGAGGATGGTGAATCCG

TTGATCCTTCCAACTTCAAGATCGACAGAAAGGATAAGGCTTTCACCAAGTACAAGGTCAAAGAGTCTCATTCCACAGAGAAAACTTGTCCCGCAAGTAA

CCTAATCAAGAAACCAATAAGAGGCCATCTTTCTGTGAAGATTAATAAAGAGAAAGAATATGATGAATGCAGAGCAGAGAAGAGATACCAAAATCAAGTT

TCCAGTGGAAGAAAATCGTCTTCAGGGATAAACCTCAGAAGAGTAACTTCACCAAGAATTCAACTCTCAGGTACGCGTACAAGCACGTCGAGATCAAAGA

GCAAACAAGTTGTTCTTGAGAGTTTTGCAGTGGTGAAGCGTTCCGTTGATCCAAAGAAAGATTTCAGAGAATCGATGGTGGAGATGATAGATGAGAACAA

CATAAGAGCTTCAAAAGACTTGGAGGATCTTCTTGCTTGTTACCTTTCCTTGAATCCAAAGGAGTATCATGATCTTATCATCCACGTTTTTGAGCAAATC

TGGCGTCAATTTACAAAAACAAAGTAA

>BraA03g028060.3C

ATGGTTCAGGAGAAGCTAGAGCAGATGATCAGAGAGACACAAGAAGCCACACACCAAGAGAAGTTAAGAGAACAAATGATGAGGAGGAGGAGAAGAAGAA

GCAAGAGTAGCATCAGTAACACTAAGTTCATAGTGATGATGGCAATGGAGAAATGTTCTTATGATCCAAGAGAGGATTTCAGAGAGTCCATGGTCGAGAT

GATTGTTGCAAACAAGATCAGAGAAGCAGATGAACTTAGAAGCCTCTTGGAGTACTATCTATCAATGAATCCTCGCGAATATCGGTCTGCCATTCTCGAG

ATCTTCTACGAGGTTTGTGCTGATTTGTTCTTGTGTTCGTAA

>BraA03g048500.3C

ATGTCTTCAAAATCTTCCTTGAGTTCTACTAAACGTCAAAATGATACTCATGAGAGTAGCAAAAGGTTACAAAGAGTATCAGCAGAAAAGGAGAACGCTA

CAACAAGATCAGCAAATATGGAGTCAAATGAGAAGTTTGAAGAGATAATGAGCAGTGTGAGGAAGAAAGTAAGAGATTTCCAAAGAGAGACAATGGATGG

AGACAAAGAAACTGTGATCATGACGCCAAGAATTCAGGTGAACAGAGATAGGCAGCAGAGATGTGAGAGACGTGATCAAAAGCTTCTCGAACAAAAGCCA

AAGAGACCAGAACAGAACACAGAGGTTAAAGTGAAGAAACCAGCGAGAAGGACAGGTACAAGTAACAGTAGAGAGACTCTTGTGGCTCACCAGTGGCAGC

ATCTCAAGGAAACAAAACTAAGAGAAGTGAAGCTGAAGGCTGACAAACAGAGGAAATCTATGTACCTAAGAAGGGAGCTAGGGACAAAAGAAAACAGCAA

GGTTAGAGTCTTTTCACCAAGATCATCCGAAAAATGCAGAGTCAAAGCTATTGAAGACTTGAAGAAGGCTAAACTGAGAGCAAAGGAGCAGGAGATGGAG

AATGAAAGCTTTGCAGTAGTGAAATGCTCGAGCGATCCTCAAAAGGACTTTAGAGATTCAATGATTGAGATGATCATGGAGAATGGTATCAATCGCCCTG

AAGAACTCAAAGAGCTTCTGGTTTGTTATCTCAGACTCAATACTGATGAGTATCATGACATGATCATCAATGTGTTTCAGCAGCACGTCGTTTACTGGTT

TATTAGTCCTTTGACTTCAGTCATGCTACATCCAGGCTTCTTGCTTACTCTTTGTGCTCTCACCTCCATAACAGTATCCCATCTTCCACTAGAGGCACAA

GTTTGA

>BraA04g021630.3C

ATGGGGAAATACAAGTTCAAAATTTCAGATATGATCTCAAATCCATGGTTTCACAAGCTCAAAGACATGACTAAGCAGTCTAAACCCAAAAGCAAACCTA

TTTCTTCTTCATCTTCCTCACACACTCATAACAAGAAGAGCCCCTCTTATGCTCCTCCTCGGCAGTCTTCAACCTCTCATTTTTCCATCAGCTTAGTAGC

TAAAAGTCCTCACCATAACTCACCAAGAAACTCTCTTCACAGAAAAAGGATGAGTGAAAGAAAGACACTTCACAAGCCATCTCTTAAACCAATCACTCCT

TTAGGTTTTAACAATAGCAAGATCAACGGTCAAGATTCATCTCACTGTGCGCTTCCAGCTCTTGAAAAGTCCCCACAGTCTTTTGAGTATAGTTTCTACG

AAAAGGAGGATGATGGATTCGTTGATCCTTCCAACTTCAAGGTGGACACAAAGAACAAAGCTTTCACCAAGTACAAGACCAAAGAGTCTAGCTCCATGGA

GAAAGCTTGTCCTGCAAGTAACCTAACCAAGACACCACTAAAAAGCCATCTTTCTGTGAAGATTAATAAAGGGAAACAAGAAGAAGATGATGAAGCATGC

AGAGCAGAGAAGAAATACAAAAAGCAAGTTTCTAGTGGAAGAAACTCTTCTGCAGGGATAAACCTAAGAAGAGTAAACTCACCCAGGATTCAACTCTCAG

GCACGCGTAGAAGCACGTCGAGATCAGAGAGCAGACAACTTGTTCTTGAAAGTTACGCGGTGATGAAGCGTTCCGTTGATCCAAAGAAAGATTTCAGAGA

ATCAATGGTGGAGATGATAGAAGAGAACAACATCAGAGCTTCAAAAGACTTGGAGGATCTTCTTGCTTGTTACCTTTCCTTGAATGCAAAGGAGTATCAT

GATTTTATCATCCAAGTTTTCGAGCAAATATGGCGTCAACTTACAAAACAATGTGAAAAAACCCACTTAATGTAA

>BraA04g025660.3C

ATGGCGAGCAAAAGCAAGAAGAAGAAGATGGTTCCTAAGACAGTCTCTGTAGTAGACATCGGATGCGGCAACTGTAAGTTCCCAACCTTGTCATCTTTTT

TCAACCGTTTCTCCAAAAAGCCCCGCCGCTACTCTTCTAACTACGGCCACTACCACTCTTCCACCACCACCACCGCCTCCTCCTCCGCTATTCCCTCCAC

CACCCATTGGTTCTCCGACAACACCTCTTCCTCCTCCGCTACACCCTCCCACGCAGCCGTCGCCGTCGAGAAAGACTCCGACGACCCTTACCTTGACTTC

CGTCAGTCTATGCTCCAGATGATTCTCGAGAACGAGATTTACTCCAAAGACGACCTCAGAGAGCTTCTCAACTGCTTTCTCTCACTCAACGAGCCTTACT

ACCACGGCATCATCATCCGCGCTTTCTCCGAAATATGGGAAGGTGTTTTCTCCGCCGCCGTCAAACGCCGTGGCGCCGTCCAAGAATCTCCGCTCGTCCG

TCATCATGGGACGTCACGTGCGTCACGTGGTTACCATAATCTCTACCACCGATCGATGTAA

>BraA04g025670.3C

ATGAAGCTCCCATTTCTAAACAAGAACAACTCTTCGTTTTCTTGTTCTTCAAATTCAAACTCAGTTTCATCATCAACAAATACCACCTCATGGCCATGGC

CTTCTTGTCATCAAAAACCTAAAACCATATCTTTCAGAGCAACCATCACTTTCACCAACCCTATCCATGACCAACACGAGGATGAGCTCGACCCACCTGA

GGGTACTGAGTCGATAGAGAGTGTGATAAAAGGGCTAAGATCATCAGAGAGACTCATCTTCGAAAGTAAAGGAGAATCCAATTCTATACTCGAAGAAGCT

ACGACTAAGCAAGAAGAACAAGAAGAAGAAGAGGAAAGTTTCATGCTCTTGTCCTTGGAATCAGACGACCCTTACTCTGATTTCAAGGGATCCATGGAGG

AGATGGTTGCGGCACACGCGCTTCACCACGATTGGAAAAGCCTCGAGAAGCTTCTCTTACAGTTCTTGAAAGTCAACGCCAAGACCAGCCATCGATACAT

TTTCGCCGCTTTTGTCGATCTCCTCTCGAACTTAACACTGCACACGAATGAACCCATCAATATCAACATTTCAAAAGAAGAAGTAGAACGCGCCACCGCC

GGAGAAGCAAGCACTTCTTGTTGTAACAGTGTGAGTCTTGGCGAGTCTCCGTTGTCTCCTTTGTCACTCTTCACGTCGTGTTCTTCTTCTTCTTCCTCCG

ATGAGACTTCCTCGACGTCCGTACGATTCTTGCCGCTGTCTTCTTTGTTAGAGATGGATGAGAAACCTAAAGACATTTTTGTTTAG

>BraA05g009320.3C

ATGAAAATCCCATTTGTAAACAAGAACCACTCTTCGTTTTCGTGTTCTTCAAATTCAGTTTCATCATCAACAAATTCCACTTCATGGGCATGGCCTTCTT

GTCATCAAAACCCTAAAACTATATCTTTCAGAGCCACCATCACTTTCACCAACCCTATCCACGAGCAAGAAGACGATGAGGTTGACCCACCTGAGATCAA

AGAGTCGATAGAGAGTGTGATAAAAGGTCTTAGATCTTCAGAGAGACTTATCTTCGAAATCAAAGGAGAATCCAACTCTATACTCGAAGAAGCTACAACT

AAGCGAGTACAAGAAGAAATAGAAGAAGAAGCAGAGGAGGAGGAAGAAGGCTTCATGCTCTTGTCCTTGGAATCAAACGACCCTTACTCAGACTTCAAGA

GATCCATGGAGGAGATGGTTGAGGCACACTCGCTTCACCACGACTGGAGAAGCCTCGAGCAGCTTCTTGTCCAGTTCTTGAAAGTCAACGCCAAGACCAG

CCATCGATACATCTTCGCCGCTTTTGTCGATCTACTCTTGAACTTACCACCAAACGCGAATGAACCCATCAGAAACAACATTGCCAACGACGACGTGGAC

GGCATTTCTGCATCATGCGCCGCCGCCGGAGAGGCAAGCACTTCTTATTGTACCAGTATAGGTCTTGGAGAATCTCCGTTATCTCCTCTGTCGTTCTACA

CGTCGTGTTCTTCGTCTTCTTCCTCCGACGAGACTTCATCCACGTCCGTACGATTCTTACCGTTGTCTTCGTTGTTAGAGATGGATGAGAAAACTAAAGA

CATTTTGGTTTAG

>BraA05g009340.3C

ATGGCGGTGAAAAGCAAGAAGAAGAAGATGGTCCTTAAGGCAGTCTCTGTTGTTGACATCGGTTGCAGCAACTGTAAGTTCCCAACCTTGACTTCTTTTT

TCAACCGTTCCTCCAAAAAGCCCCGCCTCTACTCCTCTACTTACGGCCACGGCCACTCCTCCTCCACCACTACCACCGTCTCTTCCTCCTCCAACATTCC

CTCCACAACTCACTGGTTCTCCGACAACGTCTCTTCCTCCTCCGCCGCCACACCCTCCGCCGCTGTAGCCGTCGAGAAAGACTCCGATGACCCGTACCTC

GATTTCCGCCAGTCTATGCTACAGATGATTCTCGAGAACGAGATTTACTCCAAAGACGATCTCAGGGAGCTTCTCAACTGCTTCCTCTCACTCAACGAGC

CTTACCACCACAGCATCATCATCCGCGCCTTCTCCGAAATATGGGAAGGAGTTTTCTCTGCCGCCGTCAAACGCCGTGGCAGCGTCCAAGAGTCTCCGCT

CGTCCGTCATCATGGACCGTCACGGGCCTCACGTGATTACCATAATCACCATTACTACTGA

>BraA05g012270.3C

ATGCCAAAGATCATGTGGAAGAGCCTCCATCTTTGCTTCCCGTCAAATCTCACCAAATGCTACTCTTCGCCGTGCCTTCCTCCATCGGCCGCCGCCGCTG

CTGAGGACGACGATCCCAGCCGTCCCTCTATCGTTCTCATCAACAACTTCAACCTACTCTACCACAACGACCACAACAACTACCACCACCGTGTCGTTGA

CTTACCTTCCTCATCCACCGCCACCACCTTCTCCTCTTCTGCCACGTCATCATACGAATCCGAGAGCCAAGACATTTCTCCTGAATTATCCGCCGCTTTC

GCTTCCCGTCGCTTCTTCTTCTCTTCCCCTGGCCGATCAAATGCAATCACCGACTCACCAGAAACACGGTCAAGAGAACTCTCTGATAATAGCGACAGTG

CCACGATCAAGACACCAAAGAAGACGAAGTACGACACTAGCATGAACACCACGAGGCTTCTAAGCGGAGGTTCCGCCGTGAAGCAACACGTTTACTCACC

GGATCCGTTAACTGACTTCCGACGGTCAATGCAGGAGATGATTGATGCCGCCATCGAAGCCGGAGATCTTAGCCATCCCGACGAGGGTTATGATTACTTG

AACGAGCTGCTTCTCAGTTATCTAGCGTTGAATCCAACCGACATGCACAAGTTCATCATAAGGGCTTTCTCCGACATCATGGTTTCACTCTTGTCGGAAG

AACGTCGGATATGA

>BraA05g013910.3C

ATGGGGAATCACAAGTTCAAATTTTCAGATATGATCCCAAATGCATGGTTTCACAAGCTCAAAGACATGACTAAACCCAAAAACAAACCTGTTTCTTATT

CTTCCTCAAACACTTTTAACAAGAAAAAACTCTCCTCAGATTCTCTTCCTCATAAATCTTCAGCCTCTCATTTCTCCAACAGCTTAGTAGCTAACAGTCC

TCACCACAACTCACCAAGAAATTCTACTCACAGAAAAAGGATGAGTAAAAGAAAGACACTTTACAAGCCATCTCTTAAACCAAACACTCCTCCTTTTGCA

TCTGCAGGTTTTAACAAGAGCAAGATGAATGGTCAAGATTCCTCCCACTGCCCATTTCCAGCTCTTGAAAGATCCCCTGAGTATTTTGTGTATAGTTTCT

ACGAAGAGAAGGATGATGAATTCGTCGATCATTCCAACTTTAAGATCAAAGAAAACAACAAAGCTTTCACAAAGAAAGCTTGTCCTGCAAGAAACTCAAT

CAAGAAACCGCTAAAACCCCATCTTTCTGTGAAGATTAGTAAAGAGAAAGAAGAAGATGAAGATGATGAATGCATAGCAGAGAAGAAATACCAAAAGCAA

GTCTCTAGTGGAAGAAAATCATCTGCAGGGATAAACCTCAGAAGAGTAAATTCACCTAGAATTCAACTCTCAGGTACGCGTAGAAGCACGTCTAGATCAG

AGAACAAACAAGCTGTTCTTGAGAGTTTTGCAGTGATGAAGAGTTCGGTTGATCCAAAGAAAGACTTCAGAGAATCAATGGTGGAGATGATAGAAGAGAA

CAACATCAGAGCTTCAAAAGACTTGGAGGATCTTCTTGCTTGTTACCTTTCCTTGAATCCAAAGGAGTATCATGATCTTATCATCCAGGTATTCGAGCAA

ATCTGGCGTCAACTTACAAAAACCAAGTTAAAAAAAACCCTCTTAATGTAA

>BraA06g029810.3C

ATGAAACGTTTCAAAGTAAAGATATCAAGAATCCTCTCCTTCAAATCGTGCCGTTCAAAAGATTCCTCCGACCTCCCTTTCAATCCTGTCCCTTCACTCC

CCCGTCGACCTCCTCCATCAGCTGATCCATCAACCACCGTCACAACCGTGCCACACCGTCGTCGTTCTTCTTTTAGACAACACGTGTTAACCACTTTCGG

CTGCGGCTCAAGTCGGCGACGCTCTTCCACGCCACTGGATATTTCCCGGAGGAACTCAACGTCGGTTTCTCCGCCACAGACGCCGACGTTTCAGTGGGAA

AGCGAAGGGAAATGGCACGTGATTGTTCAAGAAGATGAGGGTGAACCTCGTCCGAAAATCTACGACGGAGATGACCGTCGTCGGCGTTCAGTGAAGAAAG

AGAGACACGCACGGCGGCGAGGGAGCACTTCCTCCGCGGACGAGGAGACGGAGAGAGAGAGTCTCTTGCCATCTTCTACAAACCTCTCGCCGGAAAGTTC

CTCTTCCGGGTTGCCACGTGTCACTAGACTACGGAGAAACCCTCCCACGAGAAAAAGCGAGTCGTCTTCTTCTCCGCCACTGTCTCCGGCAAGATTGTCG

TCGTTCGTGCAGAGATTAATCCCGTGTACGGCGGCGTCAGCGGTTGCGATGGAAGGAGTGGCGGTGGTGAAAAGATCGGAGGATCCGTACGAAGATTTCA

AGGGGTCAATGATGGAGATGATAGTAGAGAAGAACATGTCTGAAATGGCTGAGCTTGAACAGCTTCTCAGCTGCTTCTTAACGCTAAACGCGAAACGCCA

CCACCGCGCGATTGTTAGAGCGTTTTCTGAGGTTTGGGTTGCTTTGTTCTCCGGTGGTAATGACGGCAGCAGGAGGTCCAGTGTTGTTGTGGCGGTGGTG

GTCCACCTTGTTGCATGTGTTGCGGCGCCCATCCATGTTGGTGAATTCCCATGGCTTGAGGTGGTGGTGGCCTTGGACCACCGAAGCTTTGTGGGTGAAG

AAGAAACTGGGAGGTGGTGGCGGCGGCATTCAGGACGGAATTGCATGGGCGGTGGAACCGCTAAGCCTGTTGTTGTTGATGCCGCTGAGGTTGCCAAGAT

GGTGGCTAAGTTTGGTAGACTTGTGGAGGTGGTAGTGGCGGTGTTGGACGTGGGACTGGGATCGGAACTGGTTGCATACCGCCATTGGCAAACGGACGCG

GTATACTCTTGA

>BraA07g002210.3C

ATGACGAAACGTTTCAAATTAAAGATAACAAGAATCCTCTCCTTCAAGTCTTGCCGTTCAAAAGATCCTTCCGATCTCCCTTTCGATCCCGTCCGTTCAT

TTCCCCGCCCATCTCCTCCACCGGCTAACCCCATAACCACCGTCACCACCGTGCCACAGCGTCGTCGTTCTTCTTTAAGACAACACGTGTTTACCACTTT

TGGCTGCGGCTCAAGTCGGAGACGCTCTTCTGCACTGTTGGATGTTTCCCGGAGGAATTCGCCGTCGTTGTCGCCGCCGCAGACGCCGACGTTTCAGTGG

GAAAGCGAGGGGAAATGGCACGTGATTGCTCAAGTAGATGAGGGAGAATACGAAACGCCTCGTCGGAAAATATACGACGGTGATGATCGCCGTCGTTCAG

TGAAGAAATACGCACGGCGGCGAGGGAGCATTTCCTCCACCGAGGAATACGAAGAAGAGACGGGGAGAGAGAGTCTCTTACCATCTTCCACAAACCTCTC

ACCGGAAAGTTCCTCTTCGGGTTTGCCACGTGTCACCAGACGACGGAGAAACCATCCGAGGAAGAAAAACACGTCGTTGGCTGTTGAGGAGAAAAGCGAG

TCGCCTTCTCCTCCACCGTCTCCGGCAAGACTGTCGTCCTTCGTGCAGAGATTAATCCCGTGTACGGCGGCGGCGCCGGTTGTGATGGAAGGAGTGGCAG

TGGTGAAGAGATCGGAGGATCCGTACGAAGACTTCAAGGGATCAATGATGGAGATGATAGTAGAGAAGAACATGTTTGAAGTTGCTGAGCTTGAGCAGCT

TCTAAGCTGCTTCTTGACGCTAAACGCGAAACGCCACCACCGCGCGATTGTCAAAGCGTTTTCTGAGGTTTGGGTTGCTTTGTTCTCCGGTGGCAATAAC

AGCAGCCGAAGGTCCAGTGTTCGACTCTCGGATTATGATGAATGTTAG

>BraA07g043220.3C

ATGCCAAACCCATTGCAGAAATCGTTGCATGGTTACCTGTCAAAGATAAAAAAAGAGACAGGGAAGCTGCAAGTGTCGAACTCATTCTCATCATCGAAGA

AGTGGGTGCTTGCTGGCTGTAAGCATCCCAAGAAGCTCTCCTTCTCCTTCAAACACAGACGACGCCCCAGCAAGACTAGATTCAACGACGACCACGTTTA

CCAGGATCCCGGTCACGCCGCCACCTTGTCCGACATAGACCGTTTCCTTGAGGAAAACTTCAAATCCCTCTGCATCAGAGATGAGGAGGGGGAGGAGGAT

GCCAAAAGCAAGGGGAAAAGGGAACAACAGTCCTCCTCCTCAGATGAAGACGACACTGATGACTATAGTCACAGATTCGAGAGGACATGGGGACCGGCCG

TCTACGACTCCCCCAAACTGCCTAGGACAGAGAGACTATCTCCACCGCCTGGATCATCGGAGGGTAGGGCCAGCATGTATACCACATCGGAGGAGGGACC

TTCCTCGTCGAGGTCCAAATCATCCAGCTTGGTGCTGCCTGAGAAGTGCATCGCGGTGCTGAGGTACACTGAAGAGCCTCAGGAGGATTTCAAGAGGTCG

ATGGTGGAGATGATGGAGTCCAAGTTAGGAGGGAGCGAGGTGGACTGGGACTTGATGGAAGAGCTTCTCTTCTGCTATCTCGATCTCAACAACAAGAAAT

CACACAAGTTCATACTCAGCGCATTCGTAGATCTCATCATCTCTCTCCGTGACAAGGAGAAGAGTATCACCAGGAAAGGCCTCGTCAGGTCGCTCAGTAC

TCGCGCCGCCAGGGAGAGGCTTAGGAAGAGGATGGCCTCCAGCGACGCCTTTCGCAACTAA

>BraA08g013140.3C

ATGATGAAATGGGGAAGAAAGAAAGTACATGTCCCTTCTTCATCTTCTTTGTCTCGTGCTCATCATGTTTCTTGGTTTTCAAAGTTGAGAGGTTCTTCTG

ACTTGAAACCTGCAAAGGAGAAGAAGCATCATGATGAAGCTAGCCAGAAGATGTCTACAAAATCTTCCTTGAGTTCTACTAAACCTGGAAATGATATTCA

TGAGAGCAGCAAAAGGTTACAGAGAGTATCAGTAGAAAAGGAGAACGTTGCAAAAAGGTCAGCAGGCATGGAGTCAAATGAGAAGTTTGAAGAGATCATG

AGCAGTGTGAAGAAGAAAGCAAGAGATTACCGAAGAGAGACGCGTGGCTTCCTAGAAGTAGAGGCAATGGATAGAGACAAAGGAGGAACTGTGATCATGA

CGCCAAGAATTCACGTGAACAGAGATAAGAAGAGACGTGACCAAAAGCTTCTCCTACAAAAACCAAAGAGATCAGAACAGGAGTCAGAGGTCAAGGTGCA

AAAACCAGCCACAAGAACATGTACAAGAAGCTACAGTAGAGAAGATTTTGTGAAGCTGAAGGAAATAAAACTAAGAGAAGTGAAGCTGAAGGCTGACCAA

CGGAGGAAGTCTATGTACCTAAGAAGGGAGCTAGGAACCAAAGAAAACAGCAATGTTAGAGTCTTTTCACCAAGAGCATGCAGAGTTAAAGCTATGGAAG

ACTTGAAGAAGGCTAAACTGAGAGCACGCGAGCACGATGGGGGAGGAATGGAGAAGGAAAGCTTTGCAGTTGCGAAATGCTCGACCGATCCTCAGAAGGA

TTTTAGAGATTCAATGGTGGAGATGATCATGGAGAATGGTATTATCAACCACCCTGAAGAACTCAAAGGGCTTCTGGTTTGTTATCTTAGACTCAATACC

AATGAATATCATGACATGATCATCAATGTGTTTCAGCAGGTGCACAGTGATTTATATTTACATTAA

>BraA09g043680.3C

ATGGGGAAGAAAATGAAGCTCCCATTTCTGAACAAGATCATTTCATATTCATCACCTTCTTCCTCTTCATGGCCATCACCGTCTTGTCATCAACAAAACC

CTAAGAAAATATTTTTCAGAGCTTTCTTCACCGTTAACAAACCTAAAAATGTCAACAAACCCGAGCCACCTTCACGCAGCTTCTCCTCCTCCTCTACCAC

CACCAACATCATGGAAAAGCCACGGGAGATAGAGTGTATAGAGAATGTGATTAGGGGACTAAAATCATCAAAAAGACTTATCTTCGAACGTAGAGGAAGA

TCAAACTCCATACTCGAAGAAGTTACTAAGGAAGAAGATTCTATAGAAGGCTTCATGCTCTTGTCCTTAGAATCAAACGACCCTTACTTGGATTTTAAGA

AATCCATGGAAAAGATGGTTGAACTACACGCTCTTCATCATGATTGGAGAAGGCTCGAGAAGCTTCTTTTCTTGTTATTGAAAGTCAACGTTAAGACAAG

CCACGAATACATCTGCGCCGCGTTTGTCGACTTGCTCTTAAACTTAGCAGTAGAAACTTCTAAAGACATCGCCCAAGAACCTCGTCCTGTCCTCGTCGAG

GAATCTCCATCGTCGCCATTATCATTCTACACGTCGTACTCGTCCTCAGATGATACCTCGTCGACGTTTGTCCGGGCCTTACCGGAGAATTTGATAGATG

AGAAGAGAAGGGACGTTGTTTGTTGTTTATCGTCGTTGTTTGAGATGGAGGAGAAGATTATAGACAATATTTATTAG

>BraA09g063630.3C

ATGAGGAACTATAAGTTCAGATTTTCAGCTATGATCCCAAGTGAATGGCTCCACAAGCTCAAGAACATGACCAAACCCAGAAAAAAACATCCTCGTCCTT

CTTCTTGTTCCTTAAACACTACCAAGAAAACAAAAGCGTCCTCTGAGTCTAATAAGTCTCTTCCTCACTCTTCAAGAACTTACTTCTCCAGTAGATCACA

CACTTCCTTAGAATCTAAGATCCTTCATAATTCACCAAGAAACTCTCTTCACGTGATAGAGAGCAAAAGAAAGACTATTTACAAGCCTTCTCCTCCTCCC

TCTTCTTCTGTATCTGCAGGCTTTAACAAGAAGAAGATCAACTTTCCTCCGAACCAAGATTCTTCTTCCTCTGCTTCTTCCAACGTCATCGACATGAATA

GCAGAGATTTCAAGAAGAGAATGTTCAAAGAGATGAAGGTGTTTGACTCAACAGAGAAAGCTTGTCCAGCAAGTAACCGAACCAAGACATCGCGTAAACC

TCATCATCTTTCAGTTAAGGTCAACAATAAAGAGAAAGAAGAAGCGTGTAGGATCAAAAAGAATGTTTCTAACGGAAGAAGATCATCATCAAACTCTCCA

AGGATAAAAATCAAAGTGAATTCTCCTAGGGTTCAAGTCCCAGCGCGTAGAAGCAAATCAAGATCGCAGAACAAACAAGTTCTTGAAAGTTTCGCAGTGA

TCAAGAGCTCTCTTGATCCGAAGAAAGATTTCAGAGAATCAATGGTGGAGATGATAGAAGAGAACAACATCAGAGCTTCACAAGACTTGAGGGAACTGCT

AGCATGTTACCTTTCATTGAATCCAAAGGAGTATCATGATCTTATCCTAAAGGTTTTCGTTCAAGTATGGCTTGAAGTCATAAACTCTAAAGTTGCGTCA

AAGTAA

>BraA09g064430.3C

ATGTGGAAAAGCTTCCATCATTGCTTCCCATCAAATCTCAATAATCCCTCCTCATCTCCGTCGGACGCCGCCGCCTCCGACGACGATCCCAACCGTCCAT

CCATTCTTCTCATCAACAACTTCAACCTCCTCTACAGCGACTCCTCCCCCACTGACCGCCCCATATCCAAGCGCCTCATCGACGCCGAACCTTCCTCCAC

CACGACATTCACTGCCTCGACCTCCACCGCCGCTTATTCTTCTTCCGCGTCCTTCGATGAATCCGATGATTACGATTTTACCCCTGAACACTCTCCCCCT

CCTGACTTAACCTCCGTTCTCGCCTCCAGTCGCTTCTTCGTCTCTTCCCCTGGCCGTTCTAACTTAATCACCGACTCGCCGGATCTCCGTCCCCGGTTTA

ACTACGAAACTGCCACTGCCACGACTACTACTAGGCTTCTCACTGGAGGAACCGCCGTGAAACAATACGTGCAATCTCCTGATCCTTACAACGACTTCAG

GCGATCGATGCAGGAGATGCTTGACGCCGTTACAGACGCAGGAGATGTTCGCCGTTACGAGTTCTTGCACGAACTGTTACTCAGTTACCTCTCATTGAAT

GCAGCAGATACACATAAGTTCATTATCAGAGCTTTCGCCGACATTCTCGTCTCTCTCTTATCGGACGGTCACCGGACAAGCTGA

>BraA10g004230.3C

ATGCCAAGAACCATGTGGAAAGACTTCCATCTTTGTTTTCCAACAAATCTCATCAAGCCCTCCTCCGAGGCCGCCACCTCCTCCCAAGAACCGAACCGGC

CATCCATTCTTCTCATCAACAACTTCAACCACCTTTACGACGATTCCACAGCCACCGGCCGCGGCATATCTAAGCCTATTATTGAAGTCATTCCACCTTC

CTCCATTACAACCGCCACGACATTCACTGCCTCCACCTCCACCTCCACCTCAACCACCGGTAACTCTTCCTCCTCCTCCTCCTTGTATGAGTCTCATAAT

TACGGTTTTGCCCCTGAAGACTCTCCCCCTCCGGACTTAACCGCCGTTCTTGCCACACGTCGCTTCTTCTTCTCTTCCCCTGGCCGTTCCAACTCAATCA

CCGATTCTCCGGATCTCCGTCCCCGGTTTGAATACAAAACTTCCACCACTACTACTGCTGCTACTAGGCTTCTAACCGGGGGAGCCGCCGTGAAACAATG

CGTGCAATCTCCTGATCCTTACAACGACTTCCGCCGATCAATGCAAGAGATGCTTGACGCCGTTACAGACGCAGGAGATGCTCGCCGTTACGAGTTCTTG

CACGAGCTGTTACTTAGCTACCTCTCATTGAATGCAGAAGATACACATAAGTTCATTATCAGAGCTTTCGCCGACGTTCTCGTATCTCTCTTATCCGACG

ATGGAGGCAGCAAACTACTCTTGGAAGAACCAACAACCAATGTAGTTTTTCAGATTACATCTCATCTAAGAAATGAGAATATCACATCTGTTGATGATCA

TCTTCATGATATCTCCAAGGACTGGCTATACTCTGAAGTTTCAACTGAATACAGGTTACATACTACAAGATATGATAAGAGGAAGAACAGTTCGATGCAA

TAG

>BraA10g016250.3C

ATGAAAGAGATGAATCAGAAAATGGGGACTCACAAGTTCAGATTTTCAGACATCATGCCTCCCTCATGGCTCTATAAGCTCAAAGGCATGAGCAGAAGTA

GCAGAAAACACCTTCCTTCTTCTCCCAAACACCTCTCCACTGATGCTTCTTCCTCAAGAAACACTCTTCGTCTTTCTTCATCTCCTTATCACCCTCAAGG

CTCTTCTTCACCCCCTAAATCTCCTTTCAAGAGAAAACTCAAACGTAAAACCGTTTACAAGCCTTCCTCTAGGCTCAAGCTCTCTTCCTCCTCCTTCAAC

CCTCATGCCCCTCTCACAACTTCTCGTAATCATCGCTCTAAACCAACCTCTCCAAATGAAATCATTCTTGAGCCTTCTCTCACTTCTTCTCGGAATCATC

GCTCTAAGTCATTCTCTGCAAATGCAATCATTGTAGAGCCTTCTCTCACTTCTTCTCGGGATCATCGCTCTAAACCATCCTCTGCAAATGCAGTCTCTGA

TTCCACCGTTGGAAGCTCATCAGATCCTCTGTCATCATCTCCTTCTGAGCAAGACTATCTTGAATCTCATTCAGTTGACGTCAAGAACAACCATTCTGTG

AAGAAGCATGTTTCTGAAGATCCTTTAGTCTCAGACAACTCAAGTCCTGTCCTGGCTGAGACTATGAAGAAACCACATTTCGAAATAAAGACGCAACAGA

AACTCAAGAAACCGAAAGCTGGTTCAACAGGTATAAGGATTCGGGCGCATTCACCAAGAATCGCCAGAAAGAAGACAAAGGGGAGAACGTCCCCACAACC

AATGAAGAAGGAAACAGCAGAGAGTTTCGCTATAGTTTTGACTTCAGTGGATCCAGAGAGAGACTTCAGAGAATCTATGGTCGAGATGATCGTCGAGAAC

AAGATGAAGGAGCAGAAAGACCTGGAAGATCTTCTTGCATGTTATCTTTCTCTGAACTCAAGCGAGTATCACGACACAATCATCAAAGCCTTCGAGAAAA

CATGGTTTCATTTGACTCACTCTATGTAA

>BraA10g019070.3C

ATGGCTAAGGAATCAATCAACCCATTCCAAGATTACAAGAACTCAATGAACCAGATGATTGACGAGAGAGATATCGAAACAGAAGATGATCTGAAGCAGC

TTCTTAGGTGTTTCTTGGACATAAACCCTCCTCCTCATCACAATCTCATCGTAAGGGCTTTTGTGGACATCTGTTCACATCTTCCGCCACCACACGACCG

CCGTGGAAAGTCACTCGGAAGACTGCTTCGTCTTTATGTCACTCTTGATAATAATGATGATGTTTGA

>BraA10g020720.3C

ATGGAGAAAAGAATGAGGCTCAGGGTTCCAAGAATAGTCCGGTCATCTCTAAGCTCTTGCCGACCACGAGACCTACACGACGTGGTCGACACTTCTGCTG

TTGCTAGCCAAACAACGTCTTCCGACAGATTCTTCCTTACCGAACCTAAAGCCAAGACTCGCGTGGACCGTCACAAACCTAAGCCTAACACGTTCTCTGC

GTTCCCACCAAACCCTTTCTACGAAGGAAGCCGTTCGTTTAGAGACTCAAAGAAGAACATCAAGACAAGGAGGAAGCAAAGATCATCTCAGTTCGCTTCT

GATTCTCTCCTCGCCTCACGTTTTAAGTCAAATGGGTCTTGGTGCTGGTCTTGCAGCGAGGACGAAGAAGAAGAGAGTGATGATAGAGATACACTCTTTT

CTTCTAGAAGCTTCTCCTCGGAGTCTTCCAAGGGGGAGAGTTTTGCGGTGGTGAAGAAGTCAGAGGATCCTTATGAGGATTTTAGGACGTCAATGGTGGA

GATGATTGTTGAGAGACAGATATTTGCAGCGGCTGAGCTACAAAAGCTTCTTCAGTGTTTTCTTTCTTTAAACTCTCTTCAACACCACAGTGTCATTGTT

CAAGTGTTTTTGGAAATTTATGCCACTTTGTTCTCTCCCTAA

>BraA10g031620.3C

ATGGGGAAGAAGAAGATGATGAAGCTCTCTTCTCTCTTCAGAGGCGTCTCCGGTGGATTACTGGCGGTTCCTCTCTGCTACAATGCCAAGACTCTCTCGT

TCCGAGTCGGAGACGACGTGATCAAGACTGTCAACTCTGTCTTCTTCGACAACAACGGAGGAGGAGATGTGTTAGAAGCCGAAACGCCTGAGTCGTGGTT

CACGAACTCGTCCGAGACGGCGAGTCACTCAACCGAGTCGGATCAAGACCTCGACGCTGAATCGTTGGAGATGGTGGTGAGAGGAGTCGTTAGATCGGAG

CGGCTGTTCTTCGATCCGGGAGTCACCTGCTCGATCCTCGAGAAATCGAAATCGGAATGCGATTCGAAATCCAAGGCCGCGGCGGCGGTTATATCGGAGG

ATCGGGATTGTAGTAGTATCTACACGCCGCCGATCGAGGACATCAGCGTCCCGGTGGCGATGGAGTCCGACGATCCGTACGGCGATTTCCGGCGATCGAT

GGAGGAGATGGTGAGGAGCCACGGCGAGCTGGCGAAGGATTGGCGGAGCTTGGAGGCGATGCTCGCGTGGTACTTGAGGATGAACGGGAAGAGGAGCCAC

GGCGTGATCGTGAGCGCATTCGTCGATCTCCTCTCGGGGCTCTCTGACTCCGGAGCCGGAGCCGGAGCGTCGTCGGTGTCTGACTCGGCTCGTTACTCAA

CCGCCGTATCTTCTTTGCCGACGTCGCCGTTATGTTCGTCGTCTCGAGGTCAGACGGAGATCGAAGAAGAAGAGAGACGGAGCTGTTAA
